# {1,1′-Bis[(pyridin-2-yl)meth­yl]-2,2′-bipiperid­yl}(perchlorato)copper(II) perchlorate

**DOI:** 10.1107/S2056989017009410

**Published:** 2017-06-30

**Authors:** Guang Yang, Elena V. Rybak-Akimova, Charles Campana

**Affiliations:** a Department of Chemistry, Tufts University, Medford, Massachusetts 02155, USA; b Bruker AXS Inc., 5465 E. Cheryl Parkway, Madison, WI 53711, USA

**Keywords:** crystal structure, copper(II) complex, amino­pyridine

## Abstract

The title complex, [Cu^II^(ClO_4_)(*meso*PYBP)](ClO_4_) {PYBP = 1,1′-bis­[(pyridin-2-yl)meth­yl]-2,2′-bipiperidyl, C_22_H_30_N_4_}, was prepared and found to crystallize with two crystallographically independent complex salt moieties. The metal atoms of the cations adopt a pseudo-square-pyramidal coordination geometry, where the tetra­dentate amino­pyridine ligands (PYBP) are wrapped around the Cu atoms in the equatorial plane. The Cu—O bonds involving an O atom of the coordinating perchlorate anion are approximately perpendicular to the plane. The two remaining non-coordinating perchlorate anions are involved in several C—H⋯O hydrogen bonds with the PYBP ligand and balance the total charge of the complex salt. The two crystallographically independent moieties are related to each other *via* a pseudo-translation along the *a*-axis direction. Exact translational symmetry is broken by (i) a difference in the conformation of one of the piperidine rings, featuring a chair conformation in one of the cations, and a sterically disfavored boat conformation in the other; and (ii) by modulation of the non-coordinating perchlorate anions.

## Abstract 2

The title complex, [Cu^II^(ClO_4_)(*meso*PYBP)](ClO_4_) {PYBP = 1,1′-bis­[(pyridin-2-yl)meth­yl]-2,2′-bipiperidyl, C_22_H_30_N_4_}, was prepared and found to crystallize with two crystallographically independent complex salt moieties. The metal atoms of the cations adopt a pseudo-square-pyramidal coordination geometry, where the tetra­dentate amino­pyridine ligands (PYBP) are wrapped around the Cu atoms in the equatorial plane.

## Chemical context   

**Figure 1:**
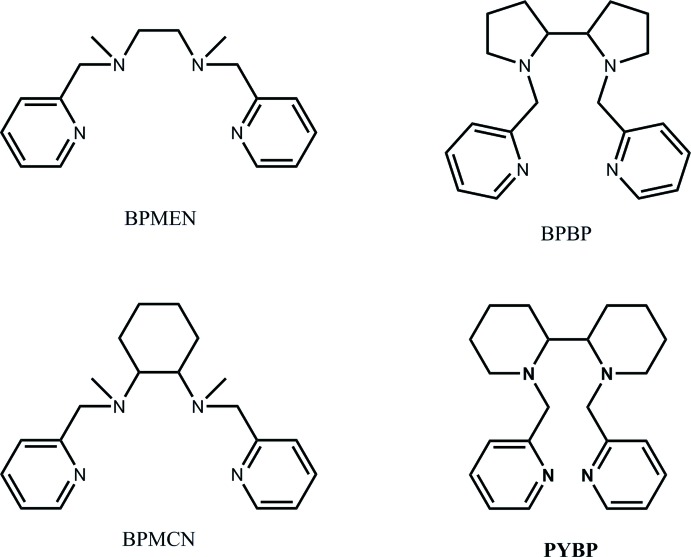
Common examples of linear tetra­dentate amino­pyridine ligands.

The design and synthesis of a family of linear tetra­dentate amino­pyridine ligands, featuring a di­amine derivative backbone (*e.g*. 1,2-cyclo­hexyldi­amine or 2,2′-dipyrrolid­yl) and two picolyl arms attached to the amine nitro­gen atoms, have frequently been discussed (Murphy & Stack, 2006 ▸; Yazerski *et al.*, 2014 ▸). Common examples of linear tetra­dentate amino­pyridine ligands are shown in Fig. 1 ▸. The Fe and Mn complexes bearing this type of ligand show good catalytic activity for olefin epoxidation (Lyakin *et al.*, 2012 ▸; Mikhalyova *et al.*, 2012 ▸), as well as aromatic (Makhlynets & Rybak-Akimova, 2010 ▸) and aliphatic (Ottenbacher *et al.*, 2015 ▸) C—H activation. Related copper(II) complexes with amino­pyridine ligands have also been synthesized and characterized (Singh *et al.*, 2017 ▸; Kani *et al.*, 2000 ▸; Liebov *et al.*, 2011 ▸). Potential applications of these complexes include fluorescent sensing of NO. The copper(II) ion in complexes with an appended fluoro­phore is readily reduced by nitric oxide with concomitant fluorescence enhancement (Kumar *et al.* 2013*a* ▸,*b* ▸).
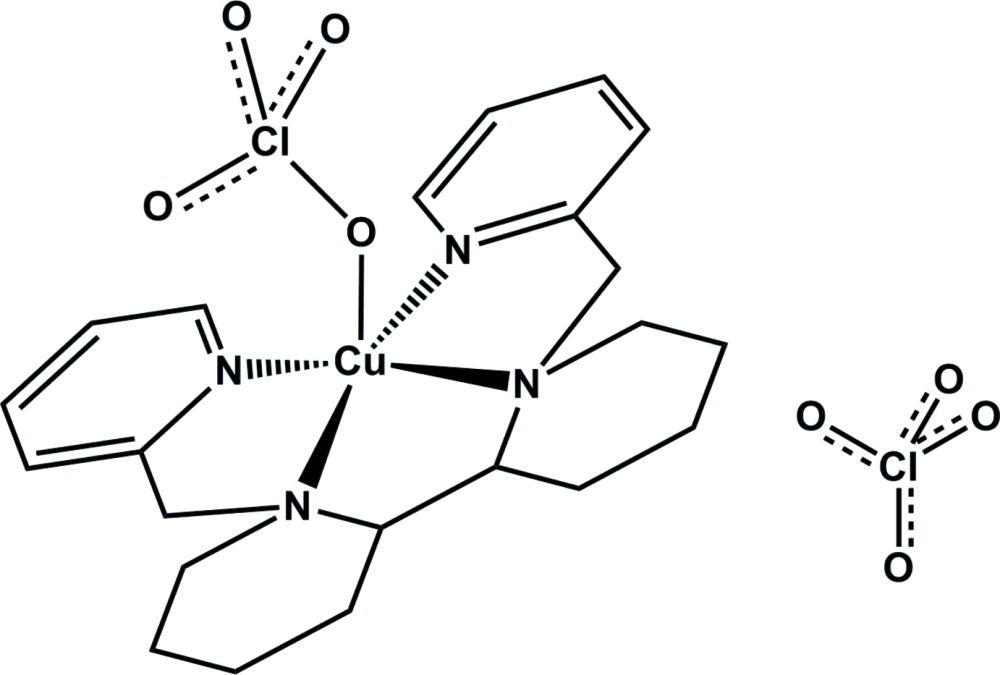


## Structural commentary   

The title compound crystallizes with two crystallographically independent moieties, consisting of a [Cu^II^(ClO_4_)(*meso*PYBP)] {PYBP = 1,1′-bis­[(pyridin-2-yl)meth­yl]-2,2′-bipiperidyl} cation and another non-coordinating ClO_4_^−^ anion [PYBP = *N*,*N*′-di-(2-picol­yl)-2,2′-dipiperid­yl]. Like some other Cu^II^ amino­pyridine complexes (Singh *et al.*, 2017 ▸; Kani *et al.*, 2000 ▸; Liebov *et al.*, 2011 ▸), the cationic complex consists of a five-coordinate Cu ion in a distorted square-pyramidal geometry.

**Figure 2:**
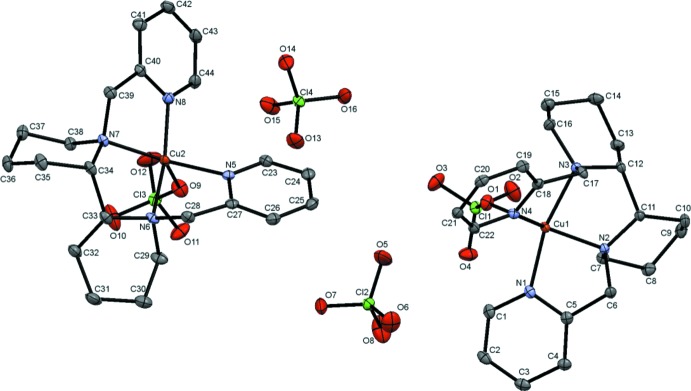
An *ORTEP* diagram of the mol­ecular structure of [Cu(*meso*PYBP)(ClO_4_)](ClO_4_), showing the atom-labeling scheme, with ellipsoids drawn at the 50% probability level. H atoms have been omitted for clarity.

**Figure 3:**
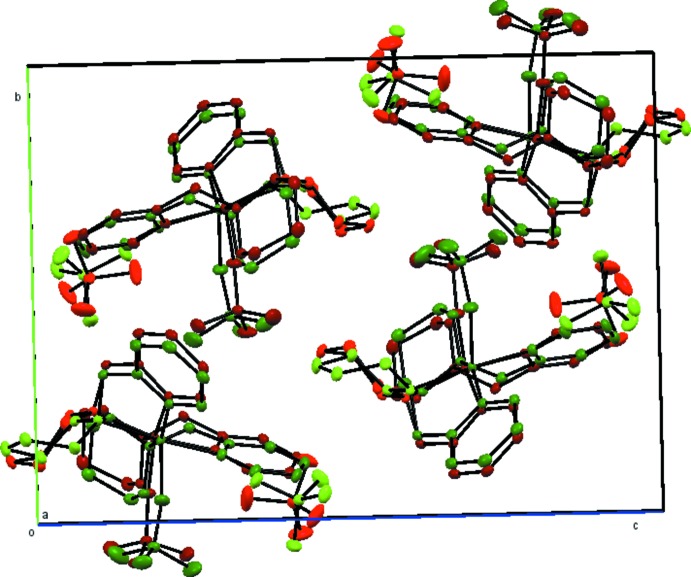
Crystal packing of the title complex viewed along *a* axis: the Cu1 (red) and Cu2 (green) moieties are related by pseudo-translation along the *a* axis. The mol­ecular parts contributing to the pseudosymmetry are highlighted. H atoms and all atom labels have been omitted for clarity.

The tetra­dentate *meso*PYBP ligand surrounds the metal ion in the basal plane (Fig. 2 ▸). One of the two remaining octa­hedral sites is occupied by the oxygen atom of a coordinating perchlorate anion, while the other site remains vacant. Another perchlorate anion in the outer sphere balances the net charge and connects nearby complex cations *via* C—H⋯O hydrogen bonds. The two chemically equivalent moieties are related to each other *via* a pseudo-translation by half a unit cell along the *a*-axis direction (Fig. 3 ▸). Similar to recently discussed crystal structures of Cu–N_2_/Py_2_ complexes (Singh, *et al.* 2017 ▸), the exact translational symmetry is broken by slightly different conformations of the two complex cations.

As shown in Fig. 3 ▸, one of the cations (the red Cu1 moiety) has both piperidine rings in a chair conformation, while the other complex cation (the green Cu2 moiety) has one piperidine ring in a sterically disfavored boat conformation (shown in light green). The reason the second cation adopts this unfavorable conformation can be tentatively traced back to the packing inter­actions of the cations and perchlorate anions. The non-coordinating perchlorate anions (shown in light red/green) are modulated along the direction of the pseudo-translation, allowing for the formation of more favorable C—H⋯O inter­actions between the C—H units of the pyridyl segments and the perchlorate oxygen atoms (see *Supra­molecular features* section), thus leading to a more favorable packing of the structure as a whole. As a result of the different conformations in the two complex cations, the Cu2—N_bp_ bonds [2.0226 (16) and 2.0078 (16) Å] differ by 0.015 Å, but the Cu2—N_py_ bonds [1.9901 (16) and 1.9890 (16) Å] are similar. In contrast, the piperidine rings of the other mol­ecule (Cu1 moiety) are both in the more favorable chair conformation; the Cu1—N_bp_ distances [2.0349 (16) and 2.0365 (16) Å] are similar, but the Cu1—N_py_ distances [1.9808 (16) and 2.0309 (16) Å] differ. These Cu—N distances fall into the range of some other Cu^II^ amino­pyridine complexes (1.98– 2.03 Å; Singh *et al.*, 2017 ▸; Kani *et al.*, 2000 ▸; Liebov *et al.*, 2011 ▸). The metal-coordinating perchlorate ions are only weakly bound, as expected for a *d* ^9^ copper(II) complex, with Cu1—O and Cu2—O distances of 2.2038 (14) and 2.3438 (15) Å, respectively.

## Supra­molecular features   

**Figure 4:**
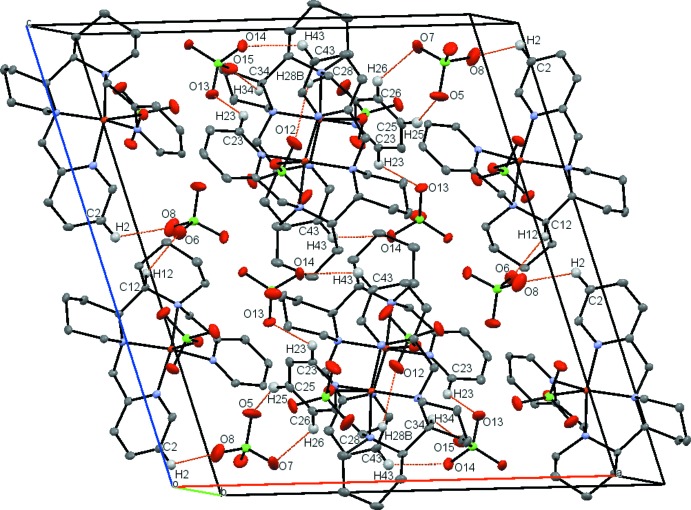
Crystal packing of the title complex viewed approximately down the *b* axis. Hydrogen bonds are shown as dashed orange lines. H atoms and all atom labels, except those involved in C—H⋯O hydrogen bonds, have been omitted for clarity.

Details of hydrogen-bonding parameters are listed in Table 1 ▸. There are in total twelve C—H⋯O hydrogen bonds, between aromatic and aliphatic C—H units and perchlorate O atoms (Fig. 4 ▸). Among these hydrogen bonds, only three involve the inner-sphere perchlorato ligand (C6—H6*A*⋯O3^ii^; C17—H17*B*⋯O4^ii^; C28—H28*B*⋯O12^iv^); all of these hydrogen bonds are inter­molecular, linking with the hydrogen atoms on the pyridine α-carbons of the adjacent Cu-*meso*PYBP cations. The perchlorate close to the Cu1 moiety forms six hydrogen bonds with four adjacent complex cations (both Cu1 and Cu2), while that close to the Cu2 moiety only forms three hydrogen bonds with two adjacent complex cations (Cu2 only). This difference in hydrogen-bonding environments of the two outer-sphere perchlorates breaks the symmetry between them and between the cation moieties. All C⋯O distances of the C—H⋯O inter­actions (3.08–3.29 Å) are roughly equal to or shorter than the sum of van der Waals radii of the corres­ponding atoms (3.25 Å), indicating normal strength inter­actions.

## Synthesis and crystallization   

The synthesis of the *meso*PYBP ligand involves two steps. A detailed synthetic procedure for (2*R*,2′*S*)-2,2′-bi­piperidine-1,1′-diium dibromide (*meso*BP·2HBr) *via* reductive hydrogenation of 2,2′-dipyridyl was reported by Herrmann *et al.* (2006 ▸) and Yang *et al.* (2013 ▸). 1.81 g *meso*BP·2HBr was dissolved in 8 mL H_2_O, and 8 mL of 5 *M* NaOH solution was added, followed by addition of 10 mL of CH_2_Cl_2_. With vigorous stirring, 4 mL of an aqueous solution containing 1.86 g picolyl chloride hydro­chloride was added dropwise, and the reaction mixture was stirred for about four days. The two layers were separated, and the aqueous layer was extracted with CH_2_Cl_2_. The organic layers were combined and the solvent was evaporated under vacuum. The ligand was purified by adding concentrated HBr and subsequent recrystallization from EtOH. ^1^H NMR (CDCl_3_): 8.60 (*d*, 2H); 8.24 (*t*, 2H); 7.80 (*d*, 2H); 7.73 (*t*, 2H); 4.25 (*d*, 2H); 3.55 (*s*, 2H); 3.07 (*d*, 2H); 2.84 (*s*, 2H); 2.05 (*d*, 2H); 1.78 (*m*, 4H); 1.66 (*m*, 4H); 1.51 (*m*, 2H). ^13^C NMR (CDCl_3_): 161.3; 149.0; 136.5; 122.5; 121.6; 63.8; 60.3; 54.2; 27.7; 24.9; 24.8. The *meso*PYBP·*x*HBr was basified with excess NaOH in aqueous solution, and extracted with CH_2_Cl_2_. The CH_2_Cl_2_ solution was dried over MgSO_4_ and solvents were removed by rotary evaporation, giving *meso*PYBP as a colorless oil (yield: 1.47g, 76%).

Under ambient atmosphere, 0.70 g (2 mmol) *meso*PYBP ligand was dissolved in 2 mL MeCN. 0.74 g (2 mmol) Cu(ClO_4_)_2_·6H_2_O (*MW* = 370.54g mol^−1^) was dissolved in minimal MeCN. The solutions were combined and stirred for two days; any precipitate was removed by filtration and discarded. 1.22 g (85%) [Cu^II^(*meso*PYBP)(ClO_4_)](ClO_4_) was obtained as dark-blue crystals by slow evaporation of the MeCN solution. The crystals decomposed and became black within 15 minutes at 503 K (caution: heating perchlorate-containing compounds may lead to explosion). UV–Vis λ_max_: 625 nm (molar absorptivity: 0.59 L mol^−1^ cm^−1^). IR (in KBr pellet) ν_max_: 3071, 2958, 1610, 1444, 1081, 1025, 780 cm^−1^.

## Refinement details   

Crystal data, data collection and structure refinement details are summarized in Table 2 ▸. H atoms were placed at calculated geometries and allowed to ride on their parent C atoms. The C—H distances were set to 0.99 Å for CH_2_, 1.00 Å for CH and 0.95 Å for aromatic CH bonds. Isotropic displacement parameters were set to 1.2 times of the equivalent isotropic displacement parameter of the parent atom.

## Supplementary Material

Crystal structure: contains datablock(s) I. DOI: 10.1107/S2056989017009410/zl2706sup1.cif

Structure factors: contains datablock(s) I. DOI: 10.1107/S2056989017009410/zl2706Isup2.hkl

CCDC reference: 1558167

Additional supporting information:  crystallographic information; 3D view; checkCIF report

## Figures and Tables

**Table 1:** Hydrogen-bond geometry (Å, °)

*D*—H⋯*A*	*D*—H	H⋯*A*	*D*⋯*A*	*D*—H⋯*A*
C2—H2⋯O8	0.95	2.62	3.259 (3)	125
C3—H3⋯O7^i^	0.95	2.55	3.265 (3)	133
C6—H6*A*⋯O3^ii^	0.99	2.58	3.259 (2)	125
C11—H11⋯O8^ii^	1.00	2.38	3.179 (3)	137
C12—H12⋯O6^iii^	1.00	2.40	3.236 (2)	141
C17—H17*B*⋯O4^ii^	0.99	2.57	3.291 (2)	130
C23—H23⋯O13	0.95	2.43	3.162 (3)	134
C25—H25⋯O5	0.95	2.31	3.164 (3)	149
C26—H26⋯O7	0.95	2.50	3.269 (3)	138
C28—H28*B*⋯O12^iv^	0.99	2.57	3.225 (3)	124
C34—H34⋯O15^iv^	1.00	2.42	3.182 (2)	132
C43—H43⋯O14	0.95	2.47	3.084 (3)	122

Symmetry codes: (i) 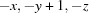; (ii) 
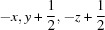
; (iii) 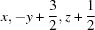; (iv) 
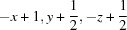
.

**Table 2:** Experimental details

Crystal data	
Chemical formula	[Cu(ClO_4_)(C_22_H_30_N_4_)]ClO_4_
*M*_r_	612.94
Crystal system, space group	Monoclinic, *P*2_1_/*c*
Temperature (K)	100
*a*, *b*, *c* (Å)	18.4079 (7), 14.0001 (5), 19.9387 (7)
β (°)	106.531 (1)
*V* (Å^3^)	4926.1 (3)
*Z*	8
Radiation type	Mo *K*α
μ (mm^−1^)	1.16
Crystal size (mm)	0.26 × 0.17 × 0.17

Data collection	
Diffractometer	Bruker APEXII CCD
Absorption correction	Multi-scan (*SADABS*; Krause *et al.*, 2015 ▸)
*T*_min_, *T*_max_	0.752, 0.832
No. of measured, independent and observed [*I* > 2σ(*I*)] reflections	144743, 12921, 9894
*R*_int_	0.062
(sin θ/λ)_max_ (Å^−1^)	0.680

Refinement	
*R*[*F* ^2^ > 2σ(*F* ^2^)], *wR*(*F* ^2^), *S*	0.038, 0.091, 1.04
No. of reflections	12921
No. of parameters	667
H-atom treatment	H-atom parameters constrained
Δρ_max_, Δρ_min_ (e Å^−3^)	0.73, −0.33

Computer programs: *APEX2* and *SAINT* (Bruker, 2013 ▸), *SHELXT* (Sheldrick, 2015*a* ▸), *SHELXL2014* (Sheldrick, 2015*b* ▸), *Mercury* (Macrae *et al.*, 2006 ▸), *OLEX2* (Dolomanov *et al.*, 2009 ▸) and *publCIF* (Westrip, 2010 ▸).

## supplementary crystallographic information

### Crystal data

**Table:**

[Cu(ClO_4_)(C_22_H_30_N_4_)]ClO_4_	*F*(000) = 2536
*M**_r_* = 612.94	*D*_x_ = 1.653 Mg m^−^^3^
Monoclinic, *P*2_1_/*c*	Mo *K*α radiation, λ = 0.71073 Å
*a* = 18.4079 (7) Å	Cell parameters from 9672 reflections
*b* = 14.0001 (5) Å	θ = 2.9–28.6°
*c* = 19.9387 (7) Å	µ = 1.16 mm^−^^1^
β = 106.531 (1)°	*T* = 100 K
*V* = 4926.1 (3) Å^3^	Block, clear dark blue
*Z* = 8	0.26 × 0.17 × 0.17 mm

### Data collection

**Table:**

Bruker APEXII CCD diffractometer	12921 independent reflections
Radiation source: sealed tube	9894 reflections with *I* > 2σ(*I*)
Graphite monochromator	*R*_int_ = 0.062
φ and ω scans	θ_max_ = 28.9°, θ_min_ = 2.7°
Absorption correction: multi-scan (SADABS; Krause *et al.*, 2015)	*h* = −24→25
*T*_min_ = 0.752, *T*_max_ = 0.832	*k* = −19→19
144743 measured reflections	*l* = −26→27

### Refinement

**Table:**

Refinement on *F*^2^	Primary atom site location: structure-invariant direct methods
Least-squares matrix: full	Secondary atom site location: difference Fourier map
*R*[*F*^2^ > 2σ(*F*^2^)] = 0.038	Hydrogen site location: inferred from neighbouring sites
*wR*(*F*^2^) = 0.091	H-atom parameters constrained
*S* = 1.04	*w* = 1/[σ^2^(*F*_o_^2^) + (0.0444*P*)^2^ + 3.2192*P*] where *P* = (*F*_o_^2^ + 2*F*_c_^2^)/3
12921 reflections	(Δ/σ)_max_ = 0.001
667 parameters	Δρ_max_ = 0.73 e Å^−^^3^
0 restraints	Δρ_min_ = −0.33 e Å^−^^3^

### Special details

**Table:**

Geometry. All esds (except the esd in the dihedral angle between two l.s. planes) are estimated using the full covariance matrix. The cell esds are taken into account individually in the estimation of esds in distances, angles and torsion angles; correlations between esds in cell parameters are only used when they are defined by crystal symmetry. An approximate (isotropic) treatment of cell esds is used for estimating esds involving l.s. planes.

### Fractional atomic coordinates and isotropic or equivalent isotropic displacement parameters (Å^2^)

**Table:**

	*x*	*y*	*z*	*U*_iso_*/*U*_eq_	
Cu1	0.02444 (2)	0.68277 (2)	0.31076 (2)	0.00959 (6)	
Cl1	0.08984 (3)	0.45819 (3)	0.32748 (2)	0.01394 (10)	
O1	0.10311 (8)	0.56114 (10)	0.32272 (8)	0.0177 (3)	
O2	0.05659 (10)	0.44218 (12)	0.38324 (9)	0.0306 (4)	
O3	0.16136 (9)	0.41064 (11)	0.34111 (9)	0.0253 (4)	
O4	0.03949 (9)	0.42629 (11)	0.26271 (8)	0.0255 (4)	
N1	−0.03644 (9)	0.65169 (12)	0.21421 (8)	0.0121 (3)	
N2	−0.07905 (9)	0.68126 (11)	0.32858 (8)	0.0105 (3)	
N3	0.06521 (9)	0.74269 (11)	0.40726 (8)	0.0102 (3)	
N4	0.10057 (9)	0.77156 (11)	0.28661 (8)	0.0109 (3)	
C1	−0.01454 (11)	0.60979 (14)	0.16256 (10)	0.0143 (4)	
H1	0.0359	0.5867	0.1720	0.017*	
C2	−0.06345 (12)	0.59934 (14)	0.09607 (10)	0.0158 (4)	
H2	−0.0465	0.5708	0.0600	0.019*	
C3	−0.13776 (12)	0.63123 (15)	0.08274 (11)	0.0163 (4)	
H3	−0.1724	0.6244	0.0375	0.020*	
C4	−0.16070 (12)	0.67310 (14)	0.13633 (10)	0.0142 (4)	
H4	−0.2113	0.6951	0.1285	0.017*	
C5	−0.10869 (11)	0.68225 (14)	0.20133 (10)	0.0128 (4)	
C6	−0.12721 (11)	0.72662 (14)	0.26330 (10)	0.0131 (4)	
H6A	−0.1174	0.7962	0.2644	0.016*	
H6B	−0.1814	0.7167	0.2597	0.016*	
C7	−0.10848 (11)	0.58239 (14)	0.33443 (10)	0.0126 (4)	
H7A	−0.0695	0.5459	0.3695	0.015*	
H7B	−0.1173	0.5495	0.2888	0.015*	
C8	−0.18186 (11)	0.58191 (15)	0.35551 (11)	0.0168 (4)	
H8A	−0.2221	0.6152	0.3195	0.020*	
H8B	−0.1984	0.5153	0.3589	0.020*	
C9	−0.16941 (12)	0.63173 (16)	0.42585 (11)	0.0185 (4)	
H9A	−0.1317	0.5957	0.4625	0.022*	
H9B	−0.2176	0.6333	0.4387	0.022*	
C10	−0.14101 (11)	0.73401 (15)	0.42194 (11)	0.0169 (4)	
H10A	−0.1262	0.7613	0.4697	0.020*	
H10B	−0.1836	0.7728	0.3933	0.020*	
C11	−0.07351 (11)	0.74408 (14)	0.39108 (10)	0.0116 (4)	
H11	−0.0760	0.8111	0.3732	0.014*	
C12	0.00505 (11)	0.73466 (14)	0.44545 (10)	0.0116 (4)	
H12	0.0113	0.7911	0.4773	0.014*	
C13	0.01545 (11)	0.64557 (15)	0.49124 (10)	0.0155 (4)	
H13A	−0.0211	0.6475	0.5192	0.019*	
H13B	0.0040	0.5884	0.4608	0.019*	
C14	0.09498 (12)	0.63660 (15)	0.54021 (11)	0.0176 (4)	
H14A	0.0987	0.5794	0.5702	0.021*	
H14B	0.1076	0.6936	0.5708	0.021*	
C15	0.14985 (12)	0.62794 (15)	0.49577 (11)	0.0183 (4)	
H15A	0.2023	0.6209	0.5264	0.022*	
H15B	0.1373	0.5708	0.4654	0.022*	
C16	0.14381 (11)	0.71641 (15)	0.45137 (11)	0.0160 (4)	
H16A	0.1763	0.7076	0.4199	0.019*	
H16B	0.1646	0.7709	0.4826	0.019*	
C17	0.07262 (11)	0.84384 (13)	0.38524 (10)	0.0114 (4)	
H17A	0.0984	0.8830	0.4265	0.014*	
H17B	0.0218	0.8714	0.3637	0.014*	
C18	0.11812 (11)	0.84388 (14)	0.33317 (10)	0.0116 (4)	
C19	0.17292 (11)	0.91093 (14)	0.33209 (10)	0.0138 (4)	
H19	0.1857	0.9597	0.3666	0.017*	
C20	0.20890 (11)	0.90567 (14)	0.27970 (10)	0.0142 (4)	
H20	0.2469	0.9507	0.2779	0.017*	
C21	0.18860 (11)	0.83379 (14)	0.23020 (10)	0.0136 (4)	
H21	0.2110	0.8304	0.1928	0.016*	
C22	0.13520 (11)	0.76678 (14)	0.23583 (10)	0.0133 (4)	
H22	0.1228	0.7161	0.2029	0.016*	
Cu2	0.49341 (2)	0.17766 (2)	0.19964 (2)	0.01095 (6)	
Cl3	0.41163 (3)	−0.05475 (3)	0.18440 (3)	0.01515 (10)	
O9	0.41362 (9)	0.04702 (10)	0.19910 (8)	0.0223 (3)	
O10	0.44337 (11)	−0.07054 (13)	0.12767 (10)	0.0412 (5)	
O11	0.33464 (9)	−0.08692 (11)	0.16571 (9)	0.0275 (4)	
O12	0.45447 (10)	−0.10456 (12)	0.24556 (9)	0.0360 (4)	
N5	0.40939 (9)	0.25668 (11)	0.21435 (8)	0.0115 (3)	
N6	0.45302 (9)	0.22413 (12)	0.09988 (8)	0.0126 (3)	
N7	0.59457 (9)	0.16475 (11)	0.18103 (8)	0.0108 (3)	
N8	0.55271 (9)	0.14352 (12)	0.29660 (9)	0.0140 (3)	
C23	0.37548 (11)	0.25160 (15)	0.26563 (10)	0.0146 (4)	
H23	0.3914	0.2041	0.3008	0.018*	
C24	0.31802 (11)	0.31360 (15)	0.26878 (11)	0.0168 (4)	
H24	0.2957	0.3097	0.3062	0.020*	
C25	0.29354 (11)	0.38124 (15)	0.21670 (11)	0.0183 (4)	
H25	0.2537	0.4239	0.2175	0.022*	
C26	0.32803 (11)	0.38601 (15)	0.16321 (11)	0.0165 (4)	
H26	0.3118	0.4315	0.1267	0.020*	
C27	0.38641 (11)	0.32336 (14)	0.16405 (10)	0.0131 (4)	
C28	0.42904 (12)	0.32362 (14)	0.10987 (10)	0.0141 (4)	
H28A	0.3962	0.3488	0.0651	0.017*	
H28B	0.4741	0.3654	0.1254	0.017*	
C29	0.38561 (12)	0.16527 (15)	0.06126 (11)	0.0187 (4)	
H29A	0.3429	0.1786	0.0809	0.022*	
H29B	0.3985	0.0967	0.0688	0.022*	
C30	0.36060 (13)	0.18549 (16)	−0.01677 (11)	0.0213 (5)	
H30A	0.3442	0.2529	−0.0249	0.026*	
H30B	0.3169	0.1443	−0.0397	0.026*	
C31	0.42578 (13)	0.16654 (17)	−0.04895 (11)	0.0239 (5)	
H31A	0.4127	0.1111	−0.0810	0.029*	
H31B	0.4322	0.2228	−0.0768	0.029*	
C32	0.50065 (12)	0.14641 (16)	0.00728 (11)	0.0194 (4)	
H32A	0.4982	0.0833	0.0289	0.023*	
H32B	0.5429	0.1452	−0.0143	0.023*	
C33	0.51477 (12)	0.22413 (14)	0.06317 (10)	0.0145 (4)	
H33	0.5098	0.2863	0.0375	0.017*	
C34	0.59262 (11)	0.22733 (14)	0.11918 (10)	0.0134 (4)	
H34	0.5980	0.2942	0.1376	0.016*	
C35	0.66139 (12)	0.21106 (15)	0.09117 (11)	0.0178 (4)	
H35A	0.6515	0.2426	0.0450	0.021*	
H35B	0.7061	0.2424	0.1233	0.021*	
C36	0.68059 (12)	0.10575 (15)	0.08316 (11)	0.0181 (4)	
H36A	0.6412	0.0771	0.0437	0.022*	
H36B	0.7297	0.1014	0.0723	0.022*	
C37	0.68517 (11)	0.05028 (15)	0.14978 (11)	0.0165 (4)	
H37A	0.7285	0.0738	0.1880	0.020*	
H37B	0.6936	−0.0183	0.1423	0.020*	
C38	0.61207 (11)	0.06226 (13)	0.17035 (10)	0.0126 (4)	
H38A	0.5696	0.0344	0.1333	0.015*	
H38B	0.6164	0.0264	0.2141	0.015*	
C39	0.64847 (11)	0.20258 (14)	0.24607 (10)	0.0143 (4)	
H39A	0.6474	0.2733	0.2454	0.017*	
H39B	0.7006	0.1816	0.2488	0.017*	
C40	0.62661 (11)	0.16657 (14)	0.30854 (10)	0.0130 (4)	
C41	0.67797 (12)	0.15686 (14)	0.37396 (11)	0.0168 (4)	
H41	0.7295	0.1746	0.3815	0.020*	
C42	0.65307 (12)	0.12073 (15)	0.42832 (11)	0.0185 (4)	
H42	0.6872	0.1140	0.4738	0.022*	
C43	0.57783 (12)	0.09455 (15)	0.41542 (11)	0.0179 (4)	
H43	0.5598	0.0688	0.4518	0.021*	
C44	0.52923 (12)	0.10627 (15)	0.34899 (10)	0.0163 (4)	
H44	0.4778	0.0875	0.3401	0.020*	
Cl2	0.13663 (3)	0.53482 (3)	0.09108 (2)	0.01590 (10)	
O5	0.17327 (11)	0.54635 (16)	0.16497 (9)	0.0475 (6)	
O6	0.10631 (10)	0.62578 (12)	0.06535 (9)	0.0315 (4)	
O7	0.19014 (9)	0.50453 (14)	0.05582 (9)	0.0357 (5)	
O8	0.07645 (11)	0.46716 (13)	0.08013 (11)	0.0414 (5)	
Cl4	0.35586 (3)	0.04176 (3)	0.41789 (2)	0.01382 (10)	
O13	0.32469 (9)	0.08657 (11)	0.35075 (8)	0.0247 (4)	
O14	0.42674 (9)	0.08605 (12)	0.45328 (9)	0.0279 (4)	
O15	0.36737 (10)	−0.05777 (11)	0.40840 (9)	0.0320 (4)	
O16	0.30413 (8)	0.05344 (12)	0.45972 (8)	0.0232 (3)	

### Atomic displacement parameters (Å^2^)

**Table:**

	*U*^11^	*U*^22^	*U*^33^	*U*^12^	*U*^13^	*U*^23^
Cu1	0.01092 (12)	0.00992 (12)	0.00883 (11)	−0.00099 (9)	0.00425 (9)	−0.00156 (9)
Cl1	0.0153 (2)	0.0108 (2)	0.0154 (2)	0.00036 (18)	0.00400 (18)	−0.00081 (18)
O1	0.0192 (8)	0.0103 (7)	0.0243 (8)	0.0011 (6)	0.0070 (6)	−0.0006 (6)
O2	0.0443 (11)	0.0272 (9)	0.0278 (9)	−0.0037 (8)	0.0222 (8)	0.0020 (7)
O3	0.0180 (8)	0.0125 (7)	0.0416 (10)	0.0043 (6)	0.0025 (7)	−0.0011 (7)
O4	0.0267 (9)	0.0212 (8)	0.0226 (8)	−0.0024 (7)	−0.0027 (7)	−0.0052 (7)
N1	0.0143 (8)	0.0110 (8)	0.0123 (8)	−0.0019 (6)	0.0058 (7)	−0.0007 (6)
N2	0.0122 (8)	0.0102 (8)	0.0099 (8)	0.0012 (6)	0.0046 (6)	0.0003 (6)
N3	0.0117 (8)	0.0098 (8)	0.0092 (8)	0.0024 (6)	0.0029 (6)	0.0012 (6)
N4	0.0115 (8)	0.0105 (8)	0.0101 (8)	−0.0004 (6)	0.0022 (6)	0.0003 (6)
C1	0.0160 (10)	0.0125 (10)	0.0165 (10)	−0.0027 (8)	0.0082 (8)	−0.0006 (8)
C2	0.0210 (11)	0.0147 (10)	0.0132 (10)	−0.0051 (8)	0.0076 (8)	−0.0022 (8)
C3	0.0205 (11)	0.0164 (10)	0.0115 (10)	−0.0055 (8)	0.0038 (8)	0.0029 (8)
C4	0.0162 (10)	0.0133 (10)	0.0127 (9)	−0.0001 (8)	0.0036 (8)	0.0030 (8)
C5	0.0151 (9)	0.0107 (9)	0.0134 (9)	−0.0003 (8)	0.0055 (8)	0.0024 (7)
C6	0.0146 (9)	0.0133 (10)	0.0115 (9)	0.0036 (8)	0.0040 (8)	0.0026 (8)
C7	0.0140 (9)	0.0115 (9)	0.0125 (9)	−0.0016 (7)	0.0040 (8)	0.0007 (7)
C8	0.0137 (10)	0.0204 (11)	0.0164 (10)	−0.0042 (8)	0.0046 (8)	0.0025 (8)
C9	0.0142 (10)	0.0261 (12)	0.0180 (11)	−0.0015 (9)	0.0091 (8)	0.0019 (9)
C10	0.0155 (10)	0.0217 (11)	0.0161 (10)	0.0042 (8)	0.0085 (8)	−0.0015 (8)
C11	0.0129 (9)	0.0118 (9)	0.0105 (9)	0.0024 (7)	0.0037 (7)	−0.0001 (7)
C12	0.0129 (9)	0.0132 (10)	0.0105 (9)	0.0002 (7)	0.0063 (7)	−0.0016 (7)
C13	0.0168 (10)	0.0194 (10)	0.0103 (9)	−0.0012 (8)	0.0039 (8)	0.0041 (8)
C14	0.0200 (11)	0.0196 (11)	0.0118 (10)	−0.0004 (9)	0.0026 (8)	0.0054 (8)
C15	0.0189 (11)	0.0187 (11)	0.0163 (10)	0.0035 (8)	0.0033 (8)	0.0018 (8)
C16	0.0117 (9)	0.0222 (11)	0.0137 (10)	0.0041 (8)	0.0030 (8)	0.0021 (8)
C17	0.0151 (9)	0.0092 (9)	0.0109 (9)	−0.0017 (7)	0.0055 (7)	−0.0018 (7)
C18	0.0121 (9)	0.0107 (9)	0.0113 (9)	0.0016 (7)	0.0024 (7)	0.0027 (7)
C19	0.0147 (10)	0.0101 (9)	0.0156 (10)	−0.0008 (8)	0.0029 (8)	−0.0010 (8)
C20	0.0114 (9)	0.0131 (10)	0.0168 (10)	−0.0012 (7)	0.0017 (8)	0.0036 (8)
C21	0.0118 (9)	0.0173 (10)	0.0121 (9)	0.0015 (8)	0.0040 (7)	0.0031 (8)
C22	0.0152 (10)	0.0141 (10)	0.0110 (9)	0.0018 (8)	0.0045 (8)	0.0007 (7)
Cu2	0.01328 (12)	0.01163 (12)	0.00875 (11)	0.00174 (9)	0.00442 (9)	0.00256 (9)
Cl3	0.0146 (2)	0.0129 (2)	0.0184 (2)	−0.00165 (18)	0.00546 (19)	0.00010 (18)
O9	0.0307 (9)	0.0131 (7)	0.0255 (8)	−0.0051 (6)	0.0117 (7)	−0.0009 (6)
O10	0.0549 (12)	0.0363 (11)	0.0472 (12)	−0.0129 (9)	0.0382 (10)	−0.0172 (9)
O11	0.0146 (8)	0.0214 (8)	0.0426 (10)	−0.0051 (6)	0.0019 (7)	0.0022 (7)
O12	0.0332 (10)	0.0255 (9)	0.0371 (10)	0.0049 (8)	−0.0098 (8)	0.0081 (8)
N5	0.0120 (8)	0.0108 (8)	0.0105 (8)	−0.0012 (6)	0.0010 (6)	−0.0008 (6)
N6	0.0169 (8)	0.0118 (8)	0.0095 (8)	−0.0039 (7)	0.0043 (7)	−0.0005 (6)
N7	0.0148 (8)	0.0085 (8)	0.0107 (8)	−0.0003 (6)	0.0061 (6)	0.0005 (6)
N8	0.0144 (8)	0.0159 (9)	0.0124 (8)	0.0028 (7)	0.0048 (7)	0.0017 (7)
C23	0.0166 (10)	0.0152 (10)	0.0118 (9)	−0.0019 (8)	0.0037 (8)	−0.0005 (8)
C24	0.0144 (10)	0.0180 (10)	0.0177 (10)	−0.0038 (8)	0.0037 (8)	−0.0042 (8)
C25	0.0119 (10)	0.0175 (10)	0.0226 (11)	0.0009 (8)	0.0003 (8)	−0.0045 (9)
C26	0.0161 (10)	0.0149 (10)	0.0159 (10)	−0.0001 (8)	0.0002 (8)	−0.0004 (8)
C27	0.0131 (9)	0.0112 (9)	0.0131 (9)	−0.0027 (7)	0.0006 (7)	−0.0016 (7)
C28	0.0187 (10)	0.0109 (9)	0.0120 (9)	0.0008 (8)	0.0035 (8)	0.0019 (7)
C29	0.0229 (11)	0.0168 (11)	0.0150 (10)	−0.0077 (8)	0.0032 (9)	−0.0015 (8)
C30	0.0250 (12)	0.0214 (11)	0.0146 (10)	−0.0032 (9)	0.0008 (9)	−0.0020 (9)
C31	0.0315 (13)	0.0264 (12)	0.0108 (10)	0.0033 (10)	0.0013 (9)	−0.0030 (9)
C32	0.0251 (11)	0.0198 (11)	0.0130 (10)	0.0022 (9)	0.0048 (9)	−0.0029 (8)
C33	0.0215 (10)	0.0122 (10)	0.0114 (9)	−0.0005 (8)	0.0070 (8)	0.0018 (8)
C34	0.0212 (10)	0.0103 (9)	0.0112 (9)	−0.0023 (8)	0.0089 (8)	0.0014 (7)
C35	0.0226 (11)	0.0158 (10)	0.0193 (11)	−0.0053 (8)	0.0128 (9)	−0.0004 (8)
C36	0.0187 (10)	0.0194 (11)	0.0208 (11)	−0.0015 (8)	0.0133 (9)	−0.0024 (9)
C37	0.0154 (10)	0.0145 (10)	0.0220 (11)	0.0000 (8)	0.0091 (8)	−0.0020 (8)
C38	0.0149 (10)	0.0093 (9)	0.0143 (10)	−0.0012 (7)	0.0056 (8)	0.0001 (7)
C39	0.0160 (10)	0.0129 (10)	0.0146 (10)	−0.0024 (8)	0.0054 (8)	−0.0013 (8)
C40	0.0166 (10)	0.0090 (9)	0.0139 (10)	0.0005 (7)	0.0049 (8)	−0.0011 (7)
C41	0.0188 (10)	0.0149 (10)	0.0161 (10)	−0.0005 (8)	0.0037 (8)	−0.0008 (8)
C42	0.0247 (11)	0.0177 (10)	0.0119 (10)	0.0058 (9)	0.0032 (8)	0.0013 (8)
C43	0.0223 (11)	0.0209 (11)	0.0128 (10)	0.0067 (9)	0.0085 (8)	0.0058 (8)
C44	0.0178 (10)	0.0173 (10)	0.0155 (10)	0.0051 (8)	0.0074 (8)	0.0049 (8)
Cl2	0.0177 (2)	0.0153 (2)	0.0144 (2)	0.00492 (19)	0.00411 (19)	0.00103 (18)
O5	0.0500 (12)	0.0691 (15)	0.0169 (9)	0.0306 (11)	−0.0009 (8)	−0.0063 (9)
O6	0.0397 (10)	0.0210 (9)	0.0356 (10)	0.0080 (7)	0.0135 (8)	0.0125 (7)
O7	0.0188 (8)	0.0560 (12)	0.0328 (10)	0.0023 (8)	0.0081 (7)	−0.0250 (9)
O8	0.0522 (12)	0.0215 (9)	0.0630 (13)	−0.0147 (8)	0.0366 (11)	−0.0078 (9)
Cl4	0.0155 (2)	0.0131 (2)	0.0130 (2)	−0.00116 (18)	0.00433 (18)	−0.00055 (18)
O13	0.0328 (9)	0.0248 (9)	0.0173 (8)	0.0013 (7)	0.0086 (7)	0.0078 (7)
O14	0.0175 (8)	0.0371 (10)	0.0300 (9)	−0.0103 (7)	0.0085 (7)	−0.0118 (8)
O15	0.0502 (11)	0.0169 (8)	0.0250 (9)	0.0078 (8)	0.0043 (8)	−0.0031 (7)
O16	0.0184 (8)	0.0351 (9)	0.0187 (8)	0.0021 (7)	0.0095 (6)	0.0066 (7)

### Geometric parameters (Å, º)

**Table:**

Cu1—O1	2.2038 (14)	Cu2—N8	1.9890 (16)
Cu1—N1	1.9808 (16)	Cl3—O9	1.4530 (15)
Cu1—N2	2.0349 (16)	Cl3—O10	1.4302 (18)
Cu1—N3	2.0365 (16)	Cl3—O11	1.4316 (15)
Cu1—N4	2.0309 (16)	Cl3—O12	1.4311 (17)
Cl1—O1	1.4695 (14)	N5—C23	1.343 (2)
Cl1—O2	1.4311 (16)	N5—C27	1.347 (3)
Cl1—O3	1.4306 (15)	N6—C28	1.491 (3)
Cl1—O4	1.4301 (15)	N6—C29	1.506 (3)
N1—C1	1.343 (2)	N6—C33	1.517 (2)
N1—C5	1.351 (3)	N7—C34	1.505 (2)
N2—C6	1.492 (2)	N7—C38	1.499 (2)
N2—C7	1.503 (2)	N7—C39	1.488 (2)
N2—C11	1.505 (2)	N8—C40	1.352 (3)
N3—C12	1.516 (2)	N8—C44	1.344 (3)
N3—C16	1.509 (2)	C23—H23	0.9500
N3—C17	1.500 (2)	C23—C24	1.383 (3)
N4—C18	1.349 (2)	C24—H24	0.9500
N4—C22	1.343 (2)	C24—C25	1.383 (3)
C1—H1	0.9500	C25—H25	0.9500
C1—C2	1.382 (3)	C25—C26	1.389 (3)
C2—H2	0.9500	C26—H26	0.9500
C2—C3	1.391 (3)	C26—C27	1.384 (3)
C3—H3	0.9500	C27—C28	1.505 (3)
C3—C4	1.386 (3)	C28—H28A	0.9900
C4—H4	0.9500	C28—H28B	0.9900
C4—C5	1.381 (3)	C29—H29A	0.9900
C5—C6	1.506 (3)	C29—H29B	0.9900
C6—H6A	0.9900	C29—C30	1.518 (3)
C6—H6B	0.9900	C30—H30A	0.9900
C7—H7A	0.9900	C30—H30B	0.9900
C7—H7B	0.9900	C30—C31	1.536 (3)
C7—C8	1.525 (3)	C31—H31A	0.9900
C8—H8A	0.9900	C31—H31B	0.9900
C8—H8B	0.9900	C31—C32	1.535 (3)
C8—C9	1.524 (3)	C32—H32A	0.9900
C9—H9A	0.9900	C32—H32B	0.9900
C9—H9B	0.9900	C32—C33	1.526 (3)
C9—C10	1.534 (3)	C33—H33	1.0000
C10—H10A	0.9900	C33—C34	1.547 (3)
C10—H10B	0.9900	C34—H34	1.0000
C10—C11	1.542 (3)	C34—C35	1.539 (3)
C11—H11	1.0000	C35—H35A	0.9900
C11—C12	1.546 (3)	C35—H35B	0.9900
C12—H12	1.0000	C35—C36	1.535 (3)
C12—C13	1.525 (3)	C36—H36A	0.9900
C13—H13A	0.9900	C36—H36B	0.9900
C13—H13B	0.9900	C36—C37	1.520 (3)
C13—C14	1.516 (3)	C37—H37A	0.9900
C14—H14A	0.9900	C37—H37B	0.9900
C14—H14B	0.9900	C37—C38	1.523 (3)
C14—C15	1.526 (3)	C38—H38A	0.9900
C15—H15A	0.9900	C38—H38B	0.9900
C15—H15B	0.9900	C39—H39A	0.9900
C15—C16	1.508 (3)	C39—H39B	0.9900
C16—H16A	0.9900	C39—C40	1.501 (3)
C16—H16B	0.9900	C40—C41	1.383 (3)
C17—H17A	0.9900	C41—H41	0.9500
C17—H17B	0.9900	C41—C42	1.387 (3)
C17—C18	1.508 (3)	C42—H42	0.9500
C18—C19	1.383 (3)	C42—C43	1.384 (3)
C19—H19	0.9500	C43—H43	0.9500
C19—C20	1.389 (3)	C43—C44	1.381 (3)
C20—H20	0.9500	C44—H44	0.9500
C20—C21	1.384 (3)	Cl2—O5	1.4442 (18)
C21—H21	0.9500	Cl2—O6	1.4260 (16)
C21—C22	1.386 (3)	Cl2—O7	1.4278 (16)
C22—H22	0.9500	Cl2—O8	1.4264 (18)
Cu2—O9	2.3438 (15)	Cl4—O13	1.4415 (15)
Cu2—N5	1.9901 (16)	Cl4—O14	1.4355 (16)
Cu2—N6	2.0226 (16)	Cl4—O15	1.4302 (16)
Cu2—N7	2.0078 (16)	Cl4—O16	1.4426 (15)
			
N1—Cu1—O1	96.20 (6)	N8—Cu2—O9	89.24 (6)
N1—Cu1—N2	82.45 (6)	N8—Cu2—N5	103.00 (7)
N1—Cu1—N3	164.41 (7)	N8—Cu2—N6	168.41 (7)
N1—Cu1—N4	98.15 (6)	N8—Cu2—N7	82.92 (7)
N2—Cu1—O1	126.30 (6)	O10—Cl3—O9	108.58 (10)
N2—Cu1—N3	87.19 (6)	O10—Cl3—O11	110.02 (11)
N3—Cu1—O1	99.32 (6)	O10—Cl3—O12	110.39 (12)
N4—Cu1—O1	91.27 (6)	O11—Cl3—O9	109.11 (10)
N4—Cu1—N2	142.29 (6)	O12—Cl3—O9	109.25 (10)
N4—Cu1—N3	83.01 (6)	O12—Cl3—O11	109.46 (11)
O2—Cl1—O1	108.63 (9)	Cl3—O9—Cu2	138.24 (10)
O3—Cl1—O1	107.93 (9)	C23—N5—Cu2	129.19 (14)
O3—Cl1—O2	110.69 (11)	C23—N5—C27	119.21 (17)
O4—Cl1—O1	108.97 (9)	C27—N5—Cu2	111.60 (13)
O4—Cl1—O2	110.01 (10)	C28—N6—Cu2	102.07 (11)
O4—Cl1—O3	110.56 (10)	C28—N6—C29	110.32 (16)
Cl1—O1—Cu1	130.44 (9)	C28—N6—C33	110.81 (15)
C1—N1—Cu1	129.13 (14)	C29—N6—Cu2	110.23 (12)
C1—N1—C5	119.14 (17)	C29—N6—C33	112.00 (15)
C5—N1—Cu1	111.68 (13)	C33—N6—Cu2	110.99 (12)
C6—N2—Cu1	101.34 (11)	C34—N7—Cu2	106.98 (12)
C6—N2—C7	108.77 (15)	C38—N7—Cu2	111.13 (12)
C6—N2—C11	110.92 (14)	C38—N7—C34	113.38 (15)
C7—N2—Cu1	113.53 (11)	C39—N7—Cu2	103.32 (11)
C7—N2—C11	114.52 (15)	C39—N7—C34	111.19 (15)
C11—N2—Cu1	106.99 (11)	C39—N7—C38	110.33 (15)
C12—N3—Cu1	108.57 (11)	C40—N8—Cu2	111.12 (13)
C16—N3—Cu1	118.99 (12)	C44—N8—Cu2	129.77 (14)
C16—N3—C12	113.91 (14)	C44—N8—C40	119.06 (17)
C17—N3—Cu1	98.75 (11)	N5—C23—H23	119.1
C17—N3—C12	110.92 (14)	N5—C23—C24	121.90 (19)
C17—N3—C16	104.40 (15)	C24—C23—H23	119.1
C18—N4—Cu1	110.03 (12)	C23—C24—H24	120.5
C22—N4—Cu1	130.98 (13)	C25—C24—C23	119.0 (2)
C22—N4—C18	118.95 (17)	C25—C24—H24	120.5
N1—C1—H1	119.1	C24—C25—H25	120.4
N1—C1—C2	121.75 (19)	C24—C25—C26	119.14 (19)
C2—C1—H1	119.1	C26—C25—H25	120.4
C1—C2—H2	120.5	C25—C26—H26	120.6
C1—C2—C3	119.08 (19)	C27—C26—C25	118.89 (19)
C3—C2—H2	120.5	C27—C26—H26	120.6
C2—C3—H3	120.4	N5—C27—C26	121.78 (19)
C4—C3—C2	119.17 (19)	N5—C27—C28	114.74 (17)
C4—C3—H3	120.4	C26—C27—C28	123.47 (18)
C3—C4—H4	120.6	N6—C28—C27	109.16 (16)
C5—C4—C3	118.75 (19)	N6—C28—H28A	109.8
C5—C4—H4	120.6	N6—C28—H28B	109.8
N1—C5—C4	122.09 (18)	C27—C28—H28A	109.8
N1—C5—C6	114.54 (17)	C27—C28—H28B	109.8
C4—C5—C6	123.37 (18)	H28A—C28—H28B	108.3
N2—C6—C5	108.85 (15)	N6—C29—H29A	109.0
N2—C6—H6A	109.9	N6—C29—H29B	109.0
N2—C6—H6B	109.9	N6—C29—C30	112.80 (17)
C5—C6—H6A	109.9	H29A—C29—H29B	107.8
C5—C6—H6B	109.9	C30—C29—H29A	109.0
H6A—C6—H6B	108.3	C30—C29—H29B	109.0
N2—C7—H7A	109.0	C29—C30—H30A	109.5
N2—C7—H7B	109.0	C29—C30—H30B	109.5
N2—C7—C8	113.12 (16)	C29—C30—C31	110.53 (18)
H7A—C7—H7B	107.8	H30A—C30—H30B	108.1
C8—C7—H7A	109.0	C31—C30—H30A	109.5
C8—C7—H7B	109.0	C31—C30—H30B	109.5
C7—C8—H8A	109.7	C30—C31—H31A	109.2
C7—C8—H8B	109.7	C30—C31—H31B	109.2
H8A—C8—H8B	108.2	H31A—C31—H31B	107.9
C9—C8—C7	109.79 (16)	C32—C31—C30	111.86 (18)
C9—C8—H8A	109.7	C32—C31—H31A	109.2
C9—C8—H8B	109.7	C32—C31—H31B	109.2
C8—C9—H9A	109.6	C31—C32—H32A	109.8
C8—C9—H9B	109.6	C31—C32—H32B	109.8
C8—C9—C10	110.16 (17)	H32A—C32—H32B	108.2
H9A—C9—H9B	108.1	C33—C32—C31	109.47 (18)
C10—C9—H9A	109.6	C33—C32—H32A	109.8
C10—C9—H9B	109.6	C33—C32—H32B	109.8
C9—C10—H10A	108.4	N6—C33—C32	110.92 (16)
C9—C10—H10B	108.4	N6—C33—H33	106.0
C9—C10—C11	115.50 (16)	N6—C33—C34	108.61 (15)
H10A—C10—H10B	107.5	C32—C33—H33	106.0
C11—C10—H10A	108.4	C32—C33—C34	118.57 (17)
C11—C10—H10B	108.4	C34—C33—H33	106.0
N2—C11—C10	113.86 (16)	N7—C34—C33	112.09 (15)
N2—C11—H11	105.5	N7—C34—H34	105.5
N2—C11—C12	111.11 (15)	N7—C34—C35	112.40 (16)
C10—C11—H11	105.5	C33—C34—H34	105.5
C10—C11—C12	114.29 (16)	C35—C34—C33	115.00 (16)
C12—C11—H11	105.5	C35—C34—H34	105.5
N3—C12—C11	108.21 (15)	C34—C35—H35A	108.6
N3—C12—H12	107.1	C34—C35—H35B	108.6
N3—C12—C13	112.09 (15)	H35A—C35—H35B	107.6
C11—C12—H12	107.1	C36—C35—C34	114.67 (16)
C13—C12—C11	114.99 (16)	C36—C35—H35A	108.6
C13—C12—H12	107.1	C36—C35—H35B	108.6
C12—C13—H13A	109.0	C35—C36—H36A	109.5
C12—C13—H13B	109.0	C35—C36—H36B	109.5
H13A—C13—H13B	107.8	H36A—C36—H36B	108.0
C14—C13—C12	112.76 (17)	C37—C36—C35	110.90 (17)
C14—C13—H13A	109.0	C37—C36—H36A	109.5
C14—C13—H13B	109.0	C37—C36—H36B	109.5
C13—C14—H14A	110.1	C36—C37—H37A	109.6
C13—C14—H14B	110.1	C36—C37—H37B	109.6
C13—C14—C15	108.06 (17)	C36—C37—C38	110.21 (17)
H14A—C14—H14B	108.4	H37A—C37—H37B	108.1
C15—C14—H14A	110.1	C38—C37—H37A	109.6
C15—C14—H14B	110.1	C38—C37—H37B	109.6
C14—C15—H15A	109.9	N7—C38—C37	112.68 (16)
C14—C15—H15B	109.9	N7—C38—H38A	109.1
H15A—C15—H15B	108.3	N7—C38—H38B	109.1
C16—C15—C14	108.97 (17)	C37—C38—H38A	109.1
C16—C15—H15A	109.9	C37—C38—H38B	109.1
C16—C15—H15B	109.9	H38A—C38—H38B	107.8
N3—C16—H16A	108.3	N7—C39—H39A	109.8
N3—C16—H16B	108.3	N7—C39—H39B	109.8
C15—C16—N3	116.14 (17)	N7—C39—C40	109.50 (16)
C15—C16—H16A	108.3	H39A—C39—H39B	108.2
C15—C16—H16B	108.3	C40—C39—H39A	109.8
H16A—C16—H16B	107.4	C40—C39—H39B	109.8
N3—C17—H17A	110.0	N8—C40—C39	115.35 (17)
N3—C17—H17B	110.0	N8—C40—C41	121.76 (18)
N3—C17—C18	108.44 (15)	C41—C40—C39	122.89 (18)
H17A—C17—H17B	108.4	C40—C41—H41	120.5
C18—C17—H17A	110.0	C40—C41—C42	118.9 (2)
C18—C17—H17B	110.0	C42—C41—H41	120.5
N4—C18—C17	113.71 (16)	C41—C42—H42	120.4
N4—C18—C19	122.12 (18)	C43—C42—C41	119.14 (19)
C19—C18—C17	124.17 (17)	C43—C42—H42	120.4
C18—C19—H19	120.6	C42—C43—H43	120.4
C18—C19—C20	118.81 (18)	C44—C43—C42	119.19 (19)
C20—C19—H19	120.6	C44—C43—H43	120.4
C19—C20—H20	120.5	N8—C44—C43	121.84 (19)
C21—C20—C19	119.02 (18)	N8—C44—H44	119.1
C21—C20—H20	120.5	C43—C44—H44	119.1
C20—C21—H21	120.4	O6—Cl2—O5	106.77 (11)
C20—C21—C22	119.21 (18)	O6—Cl2—O7	110.02 (11)
C22—C21—H21	120.4	O6—Cl2—O8	109.30 (11)
N4—C22—C21	121.80 (18)	O7—Cl2—O5	110.38 (11)
N4—C22—H22	119.1	O8—Cl2—O5	110.33 (13)
C21—C22—H22	119.1	O8—Cl2—O7	109.99 (11)
N5—Cu2—O9	85.54 (6)	O13—Cl4—O16	109.50 (9)
N5—Cu2—N6	83.25 (7)	O14—Cl4—O13	109.54 (10)
N5—Cu2—N7	151.39 (7)	O14—Cl4—O16	108.91 (9)
N6—Cu2—O9	101.07 (6)	O15—Cl4—O13	109.63 (10)
N7—Cu2—O9	122.81 (6)	O15—Cl4—O14	109.74 (11)
N7—Cu2—N6	87.02 (7)	O15—Cl4—O16	109.50 (10)
			
Cu1—N1—C1—C2	−175.28 (14)	Cu2—N5—C23—C24	178.98 (14)
Cu1—N1—C5—C4	176.50 (15)	Cu2—N5—C27—C26	179.37 (15)
Cu1—N1—C5—C6	−3.5 (2)	Cu2—N5—C27—C28	−0.6 (2)
Cu1—N2—C6—C5	46.04 (16)	Cu2—N6—C28—C27	−44.90 (16)
Cu1—N2—C7—C8	173.81 (12)	Cu2—N6—C29—C30	−170.51 (14)
Cu1—N2—C11—C10	−167.28 (13)	Cu2—N6—C33—C32	110.08 (15)
Cu1—N2—C11—C12	−36.51 (17)	Cu2—N6—C33—C34	−21.90 (18)
Cu1—N3—C12—C11	−34.01 (17)	Cu2—N7—C34—C33	−38.73 (17)
Cu1—N3—C12—C13	93.82 (15)	Cu2—N7—C34—C35	−170.07 (13)
Cu1—N3—C16—C15	−86.24 (19)	Cu2—N7—C38—C37	174.97 (13)
Cu1—N3—C17—C18	−52.50 (15)	Cu2—N7—C39—C40	41.98 (17)
Cu1—N4—C18—C17	−4.93 (19)	Cu2—N8—C40—C39	−5.7 (2)
Cu1—N4—C18—C19	175.14 (15)	Cu2—N8—C40—C41	174.63 (15)
Cu1—N4—C22—C21	−177.14 (14)	Cu2—N8—C44—C43	−174.34 (15)
O2—Cl1—O1—Cu1	−54.85 (14)	O10—Cl3—O9—Cu2	−36.13 (18)
O3—Cl1—O1—Cu1	−174.91 (11)	O11—Cl3—O9—Cu2	−156.04 (13)
O4—Cl1—O1—Cu1	64.99 (14)	O12—Cl3—O9—Cu2	84.32 (16)
N1—C1—C2—C3	−1.4 (3)	N5—C23—C24—C25	1.6 (3)
N1—C5—C6—N2	−30.3 (2)	N5—C27—C28—N6	32.0 (2)
N2—C7—C8—C9	−59.0 (2)	N6—C29—C30—C31	58.1 (2)
N2—C11—C12—N3	47.6 (2)	N6—C33—C34—N7	40.4 (2)
N2—C11—C12—C13	−78.6 (2)	N6—C33—C34—C35	170.37 (16)
N3—C12—C13—C14	52.1 (2)	N7—C34—C35—C36	45.8 (2)
N3—C17—C18—N4	40.7 (2)	N7—C39—C40—N8	−25.4 (2)
N3—C17—C18—C19	−139.32 (19)	N7—C39—C40—C41	154.29 (18)
N4—C18—C19—C20	2.5 (3)	N8—C40—C41—C42	1.3 (3)
C1—N1—C5—C4	−1.0 (3)	C23—N5—C27—C26	−0.9 (3)
C1—N1—C5—C6	179.06 (17)	C23—N5—C27—C28	179.11 (17)
C1—C2—C3—C4	0.3 (3)	C23—C24—C25—C26	−0.9 (3)
C2—C3—C4—C5	0.3 (3)	C24—C25—C26—C27	−0.6 (3)
C3—C4—C5—N1	0.0 (3)	C25—C26—C27—N5	1.5 (3)
C3—C4—C5—C6	179.92 (18)	C25—C26—C27—C28	−178.48 (18)
C4—C5—C6—N2	149.75 (18)	C26—C27—C28—N6	−147.94 (18)
C5—N1—C1—C2	1.7 (3)	C27—N5—C23—C24	−0.7 (3)
C6—N2—C7—C8	−74.19 (19)	C28—N6—C29—C30	77.5 (2)
C6—N2—C11—C10	83.02 (19)	C28—N6—C33—C32	−137.26 (17)
C6—N2—C11—C12	−146.20 (16)	C28—N6—C33—C34	90.76 (18)
C7—N2—C6—C5	−73.84 (19)	C29—N6—C28—C27	72.24 (19)
C7—N2—C11—C10	−40.5 (2)	C29—N6—C33—C32	−13.6 (2)
C7—N2—C11—C12	90.23 (19)	C29—N6—C33—C34	−145.58 (16)
C7—C8—C9—C10	57.4 (2)	C29—C30—C31—C32	−8.0 (3)
C8—C9—C10—C11	−49.8 (2)	C30—C31—C32—C33	−50.1 (2)
C9—C10—C11—N2	41.2 (2)	C31—C32—C33—N6	62.5 (2)
C9—C10—C11—C12	−88.0 (2)	C31—C32—C33—C34	−170.86 (17)
C10—C11—C12—N3	178.10 (15)	C32—C33—C34—N7	−87.4 (2)
C10—C11—C12—C13	51.9 (2)	C32—C33—C34—C35	42.6 (2)
C11—N2—C6—C5	159.35 (15)	C33—N6—C28—C27	−163.14 (15)
C11—N2—C7—C8	50.5 (2)	C33—N6—C29—C30	−46.4 (2)
C11—C12—C13—C14	176.20 (16)	C33—C34—C35—C36	−84.0 (2)
C12—N3—C16—C15	43.8 (2)	C34—N7—C38—C37	54.4 (2)
C12—N3—C17—C18	−166.32 (15)	C34—N7—C39—C40	156.41 (16)
C12—C13—C14—C15	−62.0 (2)	C34—C35—C36—C37	−50.2 (2)
C13—C14—C15—C16	60.5 (2)	C35—C36—C37—C38	54.9 (2)
C14—C15—C16—N3	−53.3 (2)	C36—C37—C38—N7	−58.0 (2)
C16—N3—C12—C11	−169.10 (15)	C38—N7—C34—C33	84.1 (2)
C16—N3—C12—C13	−41.3 (2)	C38—N7—C34—C35	−47.2 (2)
C16—N3—C17—C18	70.58 (18)	C38—N7—C39—C40	−76.89 (19)
C17—N3—C12—C11	73.46 (18)	C39—N7—C34—C33	−150.87 (16)
C17—N3—C12—C13	−158.70 (16)	C39—N7—C34—C35	77.8 (2)
C17—N3—C16—C15	164.99 (17)	C39—N7—C38—C37	−71.0 (2)
C17—C18—C19—C20	−177.39 (18)	C39—C40—C41—C42	−178.41 (19)
C18—N4—C22—C21	0.3 (3)	C40—N8—C44—C43	2.7 (3)
C18—C19—C20—C21	0.2 (3)	C40—C41—C42—C43	0.7 (3)
C19—C20—C21—C22	−2.6 (3)	C41—C42—C43—C44	−0.9 (3)
C20—C21—C22—N4	2.4 (3)	C42—C43—C44—N8	−0.8 (3)
C22—N4—C18—C17	177.15 (16)	C44—N8—C40—C39	176.75 (17)
C22—N4—C18—C19	−2.8 (3)	C44—N8—C40—C41	−2.9 (3)

### Hydrogen-bond geometry (Å, º)

**Table:**

*D*—H···*A*	*D*—H	H···*A*	*D*···*A*	*D*—H···*A*
C2—H2···O8	0.95	2.62	3.259 (3)	125
C3—H3···O7^i^	0.95	2.55	3.265 (3)	133
C6—H6*A*···O3^ii^	0.99	2.58	3.259 (2)	125
C11—H11···O8^ii^	1.00	2.38	3.179 (3)	137
C12—H12···O6^iii^	1.00	2.40	3.236 (2)	141
C17—H17*B*···O4^ii^	0.99	2.57	3.291 (2)	130
C23—H23···O13	0.95	2.43	3.162 (3)	134
C25—H25···O5	0.95	2.31	3.164 (3)	149
C26—H26···O7	0.95	2.50	3.269 (3)	138
C28—H28*B*···O12^iv^	0.99	2.57	3.225 (3)	124
C34—H34···O15^iv^	1.00	2.42	3.182 (2)	132
C43—H43···O14	0.95	2.47	3.084 (3)	122

Symmetry codes: (i) −*x*, −*y*+1, −*z*; (ii) −*x*, *y*+1/2, −*z*+1/2; (iii) *x*, −*y*+3/2, *z*+1/2; (iv) −*x*+1, *y*+1/2, −*z*+1/2.
